# Characterization of a murine model of septic cachexia

**DOI:** 10.1186/cc14061

**Published:** 2014-12-03

**Authors:** D Restagno, J-M Bonnet, K Mouchain, P Brune, C Paquet, T Delale, V Louzier

**Affiliations:** 1 VetAgro sup, campus vétérinaire, Marcy L'Etoile, France; 2 Bertin Pharma, CEA Saclay, Gig sur Yvette, France; 3 Alizé Pharma, Espace Européen, Ecully, France

## Introduction

Muscular loss is a characteristic phenomenon induced by a massive inflammation as occurring in sepsis. Indeed, the early phase of sepsis is responsible for the expression of proinflammatory cytokines, namely TNFα and IL-6, known to be effectors of cachexia. Although cachexia is a morbidity factor in human, there is to date no animal model of septic cachexia. The goal of this study is to create and characterize a murine model of septic cachexia and evaluate endogenous ghrelin variations. Among the factors involved in both sepsis and muscular protection, current research highlights a potential role for ghrelin in muscle protection but there are conflicting data regarding its variations during experimental sepsis. In this study we evaluate its two circulating forms (AG, acylated ghrelin and UAG, unacylated ghrelin).

## Methods

Sixty male C57Bl6 mice (20 to 25 g, 6 to 8 weeks) were used for these experiments. Sepsis is induced by a mild CLP (survival ≃ 80%). After anesthesia, cecum is isolated, ligated at 1/3, and punctured twice (21G needle). A laparotomy is performed without ligation or puncture. Mice weight is followed every day, blood collections and hind limb muscles dissection are realized at 2 hours, 6 hours, 1, 2, 3, 5 and 13 days after CLP. Plasmatic cytokines (TNFα, IL-6) and ghrelin levels are evaluated via the Luminex technique and ELISA assays respectively and muscles are weighed.

## Results

After surgery, weight loss in CLP mice significantly decreases from D1 to D11 with a peak at D3 (20% vs. 10% for the Sham). On D12, septic mice reach their original weight, which becomes identical to the Sham on D13. Despite a similar weight, CLP mice muscles are lighter. Circulating TNFα and IL-6 levels increase 2 hours after CLP and remain higher than for the Sham operated mice. Ghrelin concentrations, regardless of its form, increase after the surgery: AG rises on D1 and D2 while UAG increases earlier, from 2 to 24 hours.

## Conclusion

We finalize a model of muscular and weight loss due to septic conditions. Wasting syndrome and Inflammation are two of the three conditions for cachexia. The next step is to determine proteolysis in our model to confirm that we performed a murine model of septic cachexia. The role of increased circulating levels of ghrelin in this model remains to be elucidated.

